# Increased GABA_A_ Receptor ε-Subunit Expression on Ventral Respiratory Column Neurons Protects Breathing during Pregnancy

**DOI:** 10.1371/journal.pone.0030608

**Published:** 2012-01-31

**Authors:** Keith B. Hengen, Nathan R. Nelson, Kyle M. Stang, Stephen M. Johnson, Stephanie M. Crader, Jyoti J. Watters, Gordon S. Mitchell, Mary Behan

**Affiliations:** 1 Neuroscience Training Program, University of Wisconsin, Madison, Wisconsin, United States of America; 2 Department of Comparative Biomedical Sciences, University of Wisconsin, Madison, Wisconsin, United States of America; The Research Center of Neurobiology-Neurophysiology of Marseille, France

## Abstract

GABAergic signaling is essential for proper respiratory function. Potentiation of this signaling with allosteric modulators such as anesthetics, barbiturates, and neurosteroids can lead to respiratory arrest. Paradoxically, pregnant animals continue to breathe normally despite nearly 100-fold increases in circulating neurosteroids. ε subunit-containing GABA_A_Rs are insensitive to positive allosteric modulation, thus we hypothesized that pregnant rats increase ε subunit-containing GABA_A_R expression on brainstem neurons of the ventral respiratory column (VRC). *In vivo*, pregnancy rendered respiratory motor output insensitive to otherwise lethal doses of pentobarbital, a barbiturate previously used to categorize the ε subunit. Using electrode array recordings *in vitro*, we demonstrated that putative respiratory neurons of the preBötzinger Complex (preBötC) were also rendered insensitive to the effects of pentobarbital during pregnancy, but unit activity in the VRC was rapidly inhibited by the GABA_A_R agonist, muscimol. VRC unit activity from virgin and post-partum females was potently inhibited by both pentobarbital and muscimol. Brainstem ε subunit mRNA and protein levels were increased in pregnant rats, and GABA_A_R ε subunit expression co-localized with a marker of rhythm generating neurons (neurokinin 1 receptors) in the preBötC. These data support the hypothesis that pregnancy renders respiratory motor output and respiratory neuron activity insensitive to barbiturates, most likely via increased ε subunit-containing GABA_A_R expression on respiratory rhythm-generating neurons. Increased ε subunit expression may be critical to preserve respiratory function (and life) despite increased neurosteroid levels during pregnancy.

## Introduction

The control of respiration has long been considered a hard-wired, immutable neural network. Only recently has it been demonstrated that the neural control of breathing is in fact highly plastic [1]. Normal breathing requires a complex interplay of excitatory (e.g. glutamate) and inhibitory (e.g. GABA) neurotransmitters, each system providing myriad possibilities for plasticity. GABA is a critical neurotransmitter in ventilatory control, and plays key roles in rhythm generation and burst pattern formation [2]. For example, tonic GABA_A_ receptor (GABA_A_R) activation provides gain modulation of bursting activity in medullary respiratory neurons of the ventral respiratory column (VRC) [3]. Thus, due to their direct impact on the gain control of the respiratory system, allosteric modulators of these GABA_A_Rs, including anesthetics, ethanol, endogenous neurosteroids, and many anxiolytics, are often potent respiratory depressants [4]–[7].

During pregnancy, endogenous neurosteroids that inhibit breathing in non-pregnant animals [5], [6], increase up to 100-fold [8]. Yet pregnant animals breathe without difficulty [9]. The ability of pregnant females to generate respiratory rhythm implies that GABA_A_Rs are still present. We hypothesized that, during pregnancy, GABA_A_Rs on respiratory neurons are highly plastic and express GABA_A_R subtypes that are unaffected by allosteric modulators.

We previously described a similar respiratory plasticity in hibernating ground squirrels [10], [11] that enables these animals to seasonally tolerate doses of pentobarbital and ethanol far greater than would normally be lethal. The mechanism for this remarkable survival appears to be an upregulation of GABA_A_Rs containing the ε subunit. These GABA_A_Rs are insensitive to positive allosteric modulators of GABA_A_R function, including barbiturates, ethanol and neurosteroids [10], [11]. GABA_A_Rs containing ε subunits are among the only reported barbiturate-insensitive GABA_A_Rs [12], [13]. These GABA_A_R subunits are not unique to the hibernating ground squirrel; the gene for the ε subunit is highly conserved in mammals [14].

The impact of pregnancy on GABA_A_R subtype expression has been described in other regions of the brain, especially the hippocampus [15], although these investigations did not examine the ε subunit. Here, we investigated whether brainstem respiratory neurons of pregnant rats exhibit changes in GABA_A_R subunit composition similar to those observed in hibernating ground squirrels. We hypothesized that greater ε subunit expression would, as in the ground squirrel [10] and in transfected cardiac parasympathetic neurons [12], result in insensitivity to high doses of pentobarbital. We investigated the *in vivo* and *in vitro* effects of pentobarbital on respiratory function in pregnant rats to assess plasticity in ε subunit expression. We confirmed that GABA_A_R ε subunit expression is increased in respiratory neurons of the ventral respiratory column (VRC) during pregnancy in rats. Specifically, we determined if: 1) *in vivo* phrenic motor output of pregnant rats is protected against pentobarbital compared to male and virgin or post-partum female rats; 2) *in vitro* VRC respiratory neurons, including neurons of the preBötzinger complex, are protected against pentobarbital in brainstem slices from pregnant rats compared to virgin rats; and 3) brainstem expression of ε subunit-containing GABA_A_Rs is upregulated during pregnancy, particularly in the VRC.

## Methods

### Ethical approval

All experimental procedures were in accordance with NIH guidelines and this study was specifically approved by the University of Wisconsin-Madison Institutional Animal Care and Use Committee (protocol V00936).

### In vivo phrenic nerve recordings: Experimental groups

Four rat groups (Sprague Dawley, Harlan, colony 217) were studied: adult male (3–4 mo; *n* = 6), adult virgin female (3–4 mo; *n* = 6), pregnant female (G16–G18, 3–4 mo; *n* = 5), and post-partum female (30 d post-partum, 4 mo; *n* = 6). We selected G16–G18 pregnant females based on earlier studies that describe alterations in GABA_A_R subunit expression in the rat hippocampus at this gestational time point [16].

### Experimental preparation

Rats were anesthetized initially with isoflurane (3.0–3.5%, 50% O_2_) for approximately 1 h, and then slowly converted to urethane anesthesia (1.6 mg/kg, i.v.). To enable mechanical ventilation, the rats were paralyzed (pancuronium bromide, 2.5 mg/kg. i.v.), bilaterally vagotomized and ventilated with a rodent respirator (Small Animal Ventilator, Model 683, Harvard Apparatus Inc., Holliston, MA, USA). Blood samples (∼60 µl in a 125 µl capillary tube) were drawn to determine arterial blood gases (P_a_O_2_ and P_a_CO_2_), pH and base excess (ABL 810, Radiometer, Copenhagen, Denmark). Body temperature was maintained at approximately 37°C using a heated table. End-tidal CO_2_ was measured with a flow-through capnograph (Capnogard, Novametrix, Wallingford, CT, USA). The right phrenic nerve was isolated via a dorsal approach, cut distally, desheathed, submerged in mineral oil and placed on bipolar, silver wire electrodes. Nerve activity was amplified (×10,000), band pass filtered (100 Hz to 10 kHz) (Model 1700, A-M Systems, Inc., Carlsborg, WA, USA) and integrated (time constant = 50 ms, Model MA-821RSP, CWE Inc., Ardmore, PA, USA).

### Experimental protocol

**Figure 1:**
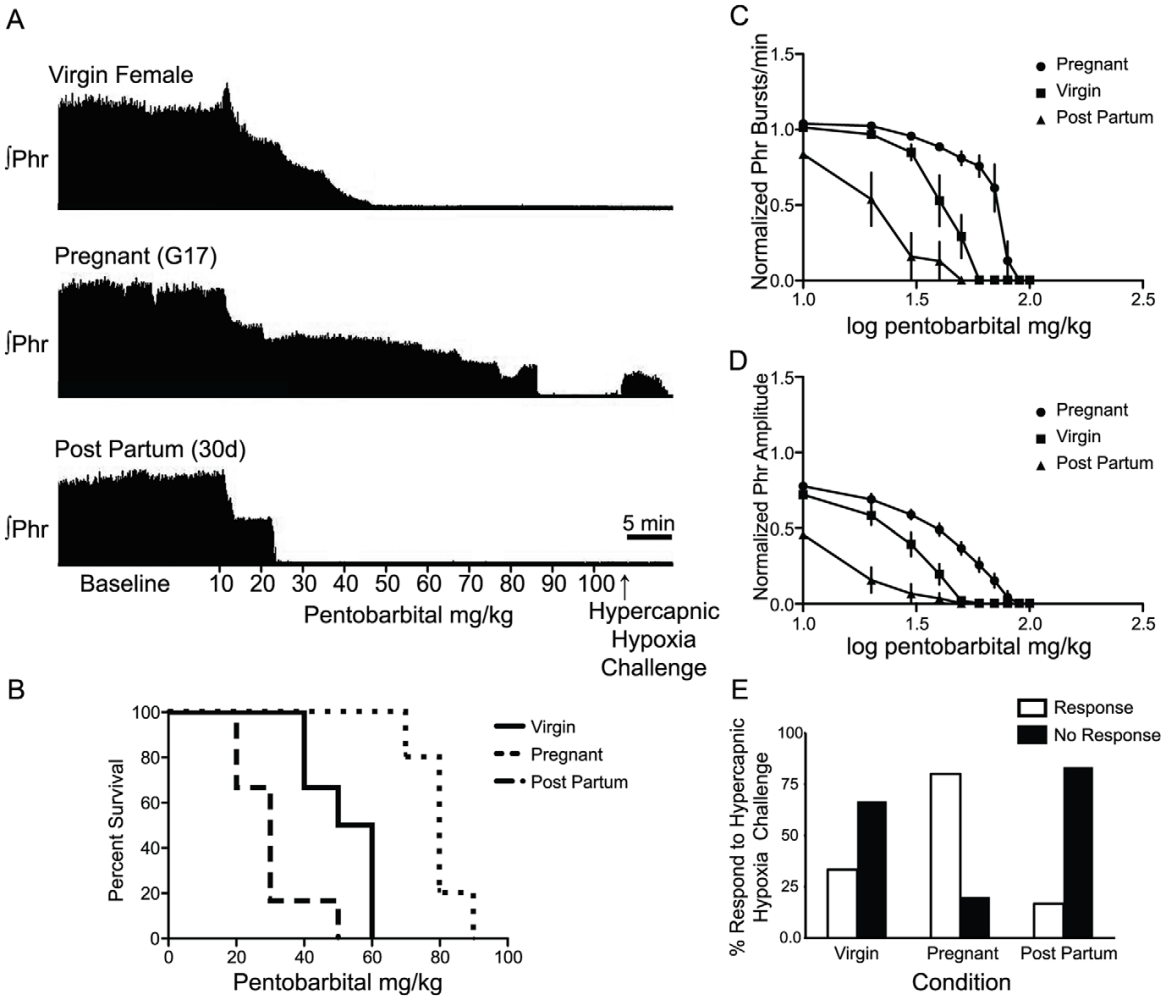
Phrenic nerve activity is insensitive to pentobarbital application during pregnancy. ***A,*** Representative, compressed phrenic neurograms from virgin female, G17 pregnant and 30 d post partum rats. After establishing baseline conditions, 10 mg/kg of pentobarbital was administered every 5 min up to a maximum total dose of 100 mg/kg. The protocol ended with 5 min of hypercapnic hypoxia to assess phrenic response to maximal chemosensory input under 100 mg/kg of pentobarbital. ***B,*** Survival plot of phrenic nerve activity by condition. 0% of post partum rats generated measurable phrenic nerve output above 50 mg/kg pentobarbital. No virgin female rats generated measureable phrenic nerve output above 60 mg/kg pentobarbital. 100% of pregnant animals generated phrenic nerve output at 60 mg/kg, and 17% generated activity at 90 mg/kg. ***C,*** Pentobarbital IC50 curve of phrenic nerve burst frequency demonstrates a significant rightward shift during pregnancy compared to virgin female and post partum animals. The hill slope of the IC50 curve during pregnancy is significantly increased as well. ***D,*** Pentobarbital IC50 curve of phrenic nerve amplitude demonstrates a significant rightward shift during pregnancy compared to virgin female and post partum animals. The hill slope of the amplitude IC50 during pregnancy is not significantly altered. ***E,*** Hypercapnic hypoxia produced a measurable response in the phrenic nerve in a greater number of pregnant animals (4/5) than virgin female (2/6), post partum (1/6), or male (0/6; not shown).

Recordings began approximately 60 min post-surgery. The nerve was positioned on the electrode and allowed to stabilize under baseline conditions of hyperoxia (P_a_O_2_>150 mmHg) and hypercapnia (P_a_CO_2_ = 60 mmHg). Hypercapnia was maintained throughout an experiment to assure a potent chemical drive to breathing. Typical tracings of phrenic motor output from a virgin female rat, a pregnant rat and a post-partum rat are shown in Fig. 1. The protocol consisted of 10 pentobarbital injections (10 mg/kg/injection i.v.), each separated by 5 min. Following pentobarbital injections, 5 min of hypercapnic hypoxia was administered (PET CO_2_>80 mmHg, 13% inspired O_2_) to estimate the scope of phrenic nerve activity. At the end of each experiment, rats were euthanized with a urethane overdose (i.v.).

### Data analysis

Phrenic nerve activity was recorded throughout the protocol. The change in peak integrated amplitude from baseline (ΔPhr), burst frequency (bursts/min), and mean arterial blood pressure (MAP) were measured at the following time points: baseline and the last minute before successive doses of pentobarbital were given. Nerve activity was averaged over that minute. Changes in burst frequency and amplitude were analyzed as raw values and normalized to baseline data. Normalization did not alter the fundamental effects of time, pentobarbital dose or condition. For statistical analyses, data were analyzed in the statistical program, R (R: A Language and Environment for Statistical Computing, R Foundation for Statistical Computing, Vienna, Austria); a mixed effect linear model (function “lmer” in package “lme4”) was used with the fixed effects being condition, dose and their interaction. The random effect was rat. Overall group differences were tested with an F test. Subsequent *post hoc* comparisons were performed with a Wald T-test. Differences were considered significant if p<0.05. All data are reported as means ± S.E.M. To analyze contingency tables, a Fisher's exact test was performed.

### In vitro recordings: Experimental groups

A total of 33 animals were used in brain slice electrophysiology studies. Seven groups of rats were studied: adult male (3–4 mo; *n* = 6), adult virgin female (3–4 mo; *n* = 6), adult male and female oxygen controls (3–4 mo; n = 4), adult male and female time controls (3–4 mo; n = 5), adult male and female bicuculline controls (3–4 mo; n = 4), pregnant female (G16–G17, 3–4 mo; *n* = 4), 30 d post-partum female (4 mo; n = 4).

### Experimental protocol

**Figure 2:**
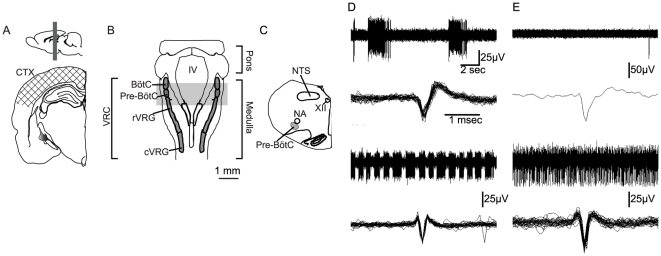
Location of slices for electrophysiological recordings and immunohistochemical analyses. Spontaneous neuronal activity was recorded in cortical slices *(* ***A*** *)*, and medullary slices *(* ***B*** *)* containing the rostral ventral respiratory group (rVRG), Pre Bötzinger (preBötC) and Bötzinger Complex (BötC) *(* ***C*** *)*. Electrode arrays placed in cortical slices were centered on primary motor/somatosensory cortex (***A***; hatched area). Electrode arrays in medullary slices were centered on the region containing the ventral respiratory column (VRC) *(* ***B*** *)*. The gray bar indicates the rostrocaudal extent of coronal sections used for *in vitro* electrophysiology and well as immunohistochemistry. During the initial equilibration, medullary neurons exhibited rhythmic bursts *(* ***D top, bottom*** *)*, slow tonic activity *(* ***E top*** *)* or fast tonic activity *(* ***E bottom*** *)*. Raw traces are shown above overlaid action potential waveforms extracted from raw data. (***D, E***). NA, nucleus ambiguus; IV, fourth ventricle. NTS, nucleus of the solitary tract; XII, hypoglossal nucleus; cVRG, caudal ventral respiratory group; rVRG, rostral ventral respiratory group; Pre-BötC, pre-Bötzinger complex; BötC, Bötzinger complex.

Brains were removed and coronal medullary and cortical slices were cut in ice-cold 3 mM KCl aCSF with a vibrating microtome (Campden Instruments, Layfayette, IN). Cortical slices (375 µm thick) contained primary motor and primary somatosensory areas. To remove the medulla, transverse cuts were made at caudally at C1 and rostrally at the pontomedullary junction. A series of slices (375 µm thick) were made through the medulla from the pontomedullary junction to the obex. The first slice used for recording was located immediately rostral to the obex and contained the rostral ventral respiratory column [17], [18]. The next slice used for recording contained the preBötzinger complex. In this slice, the hypoglossal nuclei were separated at the midline and, the caudal extremity of the nucleus ambiguus (subcompact nucleus ambiguus) was visible. The third, and final slice used for recording was adjacent and contained the Bötzinger complex. In this slice, the compact nucleus ambiguus was visible, caudal to the retrofacial nucleus [19], [20], [21] (Fig. 2). Upon removal, slices were immediately placed into an interface recording chamber (Warner Instruments, Hamden, CT) and subfused with warm artificial cerebrospinal fluid (aCSF, 37°C) at a rate of 8 ml/min. Slices were maintained at 37°C by an automated temperature controller (Harvard Apparatus, Holliston, MA). The composition of the aCSF was (in mM): 120 NaCl, 26 NaHCO_3_, 20 glucose, 2 MgSO_4_, 1.0 CaCl_2_, 1.25 Na_2_HPO_4_, 7 mM KCl.

Spontaneous activity was recorded in medullary and cortical slices from the same rat. Three 16-channel extracellular electrodes arrays (model a4x4-3mm100-177, Neuronexus, Ann Arbor, MI) were placed in three slices through VRC, ventrolateral to the nucleus ambiguus (Fig. 2). Arrays were composed of 4 shanks, each with four recording sites. The distance between each shank was 125 µm; the distance between each recording site was 75 µm, and each individual recording site had a diameter of 15 µm. Arrays were inserted into medullary slices at a 45° angle such that the top of the array touched the ventral border of the subcompact nucleus ambiguus. The array spanned the entire VRC and extended into tissue immediately adjacent. One array was inserted perpendicular to the cortical layers, centered on layer 3. Using this approach, multiple neurons (up to 25 neurons) were recorded from each of the three medullary slices and the cortical slice obtained from each animal in each condition. Slices were allowed to equilibrate in 7 mM KCl aCSF at 37°C with electrodes inserted for 60 min. Baseline activity was then measured for 30 min, followed by the application of 300 µM pentobarbital in aCSF for 60 min. To examine the contribution of GABA_A_Rs to the neuronal response to pentobarbital, a subset of slices was treated with bicuculline (100 µM, Tocris Bioscience, Ellisville, MO), a selective GABA_A_R antagonist, prior to pentobarbital application (100 µM bicuculline with 300 µM pentobarbital). To confirm the presence of pentobarbital insensitive GABA_A_Rs in slices from pregnant rats, 60 min of 300 µM pentobarbital was immediately followed by 300 µM pentobarbital and 20 µM muscimol (Tocris), a GABA_A_R agonist, for 15 min.

### Data analysis

Raw data were processed as described previously [10]. Individual neurons were identified using Principal Component Analysis [22]. Neuronal activity was averaged in 5 min bins and normalized to the mean firing rate during the 30 min baseline recording prior to drug application. Waveforms that were recorded on multiple, adjacent channels were counted only once. Neuronal waveforms were discarded from analysis if their mean baseline firing rate was ≤0.01 Hz, if they were silent for >10 consecutive min during the 1 h baseline, or if their firing rate was consistently decreasing during the 1 h baseline more than 50% of the normalized value. Individual bins were discarded if the absolute firing rate was >500 Hz, or if traces exhibited evidence of mechanical disturbances (i.e. normalized firing rate increased and then decreased more than 50 standard deviations from the baseline mean in <3 min). One cell was discarded on the basis of a constantly increasing baseline and a Grubb's outlier test. Based on these criteria, ∼10% of waveforms and <2% of data bins were discarded. To examine possible artifacts due to drift across the duration of recordings, time control experiments were run for 240 min with no drug application. Time control experiments were analyzed with Wald T-tests to test for an individual effect of time (R Foundation for Statistical Computing, Vienna, Austria).

For statistical analyses, R was used with the function “lmer” in package “lme4”. Data were analyzed with a mixed effect linear model where fixed effects were condition, time and their interaction. Random effects were rat and neurons nested within rats. Group differences were tested with an F test. Subsequent *post hoc* comparisons were performed with a Wald T-test. Differences were considered significant if p<0.05. The total number of neurons per condition was used as the number of independent samples for relevant statistical tests and calculation of S.E.M. All data are reported as means ± S.E.M.

### Real time PCR: Experimental groups

Four groups of rats (Sprague Dawley, Harlan, colony 217) were studied: adult male (3–4 mo; *n* = 6), adult virgin female (3–4 mo; *n* = 6), pregnant female (G16–G18, 3–4 mo; *n* = 6), and post-partum female (30 d post-partum, 4 mo; *n* = 5).

### Experimental protocol

Samples of the whole medulla were harvested from rats in all groups. Tissue was homogenized in Tri- Reagent (Sigma, St. Louis, MO), and total RNA was harvested according to the manufacturer's protocol. Reverse transcription PCR (RT-PCR) was performed using 1 mg of total RNA as a template for the reverse transcription reaction using random hexamers and ImProm-II Reverse Transcriptase (Promega, Madison, WI) according to the manufacturer's instructions. Quantitative RT-PCR was conducted by monitoring in real-time the increase in fluorescence of the SYBR-GREEN dye using the TaqMan 7300 Sequence Detection System (Applied Biosystems, Carlsbad, CA). The relative amounts of each gene were determined using the comparative C_T_ method [23]. C_T_ values were normalized to the relative levels of 18s in each sample.

Primer sequences for the following *rattus norvegicus* genes were used:

18s F: 5′ AAC GAG ACT CTC GGC ATG CTA A 3′

18s R: 5′ CCG GAC ATC TAA GGG CAT CA 3′

Epsilon F: 5′ TGG AGC CTC AGC CTA GTG GAA AGA 3′

Epsilon R: 5′ GGC GCA GTT TAT GGT CGT AGT TGC 3′

### Data analysis

Delta C_T_ values were used for statistical analyses. For statistical analyses, data were analyzed in R with a linear model where the fixed effect was condition. For analysis of variance, function “lme” in package “nlme” was used (R foundation for statistical computing, Vienna, Austria). *Post hoc* analyses were conducted with a Tukey's HSD test.

### Semi-quantitative immunohistochemistry: Experimental groups

Four rat groups (Sprague Dawley, Harlan, colony 217) were studied: adult male (3–4 mo; *n* = 4), adult virgin female (3–4 mo; *n* = 4), pregnant female (G16–G18, 3–4 mo; *n* = 4) and post-partum female (30 d post-partum, 4 mo; *n* = 4). These 16 animals were in addition to those used for *in vivo* and *in vitro* physiology experiments. All animals were perfused at the same time, and sections reacted simultaneously to avoid complications related to differential tissue processing.

### Tissue collection

Rats were deeply anesthetized with 5% isoflurane and transcardially perfused with 100 ml of cold saline followed by 500 ml of cold paraformaldehyde (4%). Brains were removed, post-fixed for 1 h in 4% paraformaldehyde, placed in a sucrose solution for 48 h, frozen and sectioned coronally (30 µm) on a sliding freezing microtome.

Medullary sections were harvested from identical landmarks as described above in *In Vitro* Experimental Protocol. Two matched sections from each rat, one through the rVRG and one through preBötC, were selected [21]. Control sections containing the preBötC were selected from rats in each experimental group. All sections were reacted simultaneously. Sections were washed extensively in 0.01 M PBS. After 1 h in blocking solution (10% NDS in 0.01 M PBS), a primary antibody against GABA_A_ ε (1∶1000; Abcam, ab35971, Cambridge, MA) was applied overnight at room temperature in blocking solution and 0.3% Triton X-100. Sections were exposed to a fluorescent secondary antibody (1∶300; Invitrogen, Carlsbad, CA), mounted and coverslipped with Vectashield Hard Set mounting medium for fluorescence (Vector Laboratories, Burlingame, CA). Images of the entire slice were acquired at 4× and 10×. Images of ventrolateral quadrant were acquired, bilaterally, at 40× (in preBötC slices, the sub compact nucleus ambiguus was in the upper corner of the frame). There were no labeled cells in negative control slices from all conditions.

### Colocalization immunohistochemistry: Tissue collection

Brain slices (375 µm) used for *in vitro* recordings (see above) were collected from male, virgin female, pregnant, and post-partum rats and immersion fixed in 4% paraformaldehyde for 24 h. Fixed sections were cryoprotected with a 30% sucrose solution and sectioned coronally (30 µm) on a freezing microtome. Sections were selected from each subregion of the VRC (rVRG, preBötC, BötC; [21]) and reacted simultaneously. A tyramine signal amplification kit (Perkin Elmer, Waltham, MA) was used to diminish false-positive label between antibodies. Sections were treated according to manufacturer's recommendations. A tyramine amplified primary antibody against NK1-R (1∶30,000; Sigma, S8305) was applied. A biotin-SP-conjugated secondary was applied (1∶200; Jackson Immunoresearch, West Grove, PA) prior to the tyramine reagent (Perkin Elmer, Waltham, MA). Sections were then washed and blocked again, before the application of a primary antibody against GABA_A_ ε as described above (1∶1000; Abcam 35971, produced against a synthetic peptide immunogen corresponding to human GABA_A_R ε subunit intracellular amino acids 237–286). Negative controls included sections reacted without primary antibodies, sections reacted without secondary antibodies, and sections reacted without tyramine signal amplification of the NK1-R primary antibody. Negative controls demonstrated specificity of antibody labeling. There were no labeled cells in control sections lacking primary antibodies.

### Image acquisition and analysis

All images were acquired during the same session using an Olympus Fluoview 500 laser-scanning confocal system (Tokyo, Japan) mounted on an AX-70 upright microscope. Images were analyzed using Image J software (W. Rasband, National Institutes of Health, Bethesda, MD). For colocalization histochemistry, images were scanned with different wavelengths sequentially to prevent bleed-through. User-defined thresholds were applied uniformly to all images to measure the average pixel intensity, the number, and the area of particles. Total and average fluorescence was measured on cell-by-cell basis. Total intensity was calculated as the mean cell intensity times the number of cells. All positive cells more than 300 µm from the ventral edge of the slice were quantified. For quantitation, background fluorescence measured in negative control sections was subtracted from measurement of positive label. Data were normalized to the mean expression of label in virgin female rats. For figures, 40× images were uniformly processed in Adobe Photoshop (Adobe Systems Incorporated, San Jose, CA) as follows: brightness levels adjusted, unsharp mask routine to improve edge detection, converted to 8-bit depth, and cropped.

Images (350×350 µm) were taken in the anatomical center of the VRC bilaterally; position was based on anatomical landmarks with reference to the nucleus ambiguus (compact; cNA, or subcompact; scNA), which were clearly visible under brightfield conditions and demonstrated GABA_A_ ε immunoreactivity. The viewfinder was centered ventral to the NA, one third of the distance between the ventral edge of the slice and the NA. Consistent microscope and laser settings were applied for the collection of each image.

For statistical analyses of label intensity as assessed by Image J, R was used with function “lmer” in package “lme4” (R foundation for statistical computing, Vienna, Austria). Data were analyzed with a mixed effect linear model where the fixed effect was condition. The random effects were rats and images nested within animals. Overall group differences were tested with an F test. Subsequent *post hoc* comparisons were performed with a Wald T-test. Differences were considered significant if p<0.05. Data are reported as means ± S.E.M.

## Results

### Phrenic nerve recordings in anesthetized rats

Responses of phrenic nerve activity to cumulative pentobarbital doses were assessed in: virgin female, male, pregnant female, and post-partum female rats. Phrenic nerve activity became undetectable in all virgin females, at or before a cumulative dose of 60 mg/kg pentobarbital, whereas all pregnant females ceased phrenic nerve bursting at or before a cumulative dose of 90 mg/kg. During pregnancy, rats continued to generate rhythmic phrenic motor output at significantly higher pentobarbital doses than either virgin female or post-partum rats (p<0.05) (Fig. 1*A*). Changes in pentobarbital sensitivity during pregnancy are reversible, since all post-partum females ceased bursting at cumulative pentobarbital doses below 50 mg/kg (Fig. 1*B*). The response of male rats was indistinguishable from that of virgin female rats (see *Phrenic nerve burst frequency and amplitude* in the discussion).

### Pregnancy protects rhythm generation vs. pattern formation

Phrenic burst frequency and integrated amplitude are key metrics of respiratory control, representing the processes of respiratory rhythm generation and burst pattern formation, respectively [1]. Although baseline phrenic burst frequency did not differ between groups (p = 0.44), the pentobarbital IC_50_ (dose required to inhibit a nerve activity by half) was nearly double in pregnant rats (69.3 mg/kg; 95% CI, 66.2 to 72.5 mg/kg; p<0.05) relative to virgin (40.7 mg/kg; 95% CI, 38.1 to 43.6 mg/kg) and post-partum rats (19.5 mg/kg; 95% CI, 16.5 to 23.1 mg/kg)(Fig. 1*C*). The increase in IC_50_ was accompanied by a significant increase in the hill slope of burst frequency dose/response curve in pregnant females (−9.4±1.8; p<0.01) *vs.* virgin (−6.2±1.1) or post-partum rats (−3.2±0.7).

### Pentobarbital inhibits phrenic burst amplitude in all conditions

The pentobarbital IC_50_ of phrenic burst amplitude was increased in pregnant *vs.* virgin and post-partum females (p<0.05). There are 2 possible explanations for this, (1) phrenic burst amplitude is less sensitive to pentobarbital inhibition during pregnancy, or (2) the baseline phrenic nerve amplitude is increased during pregnancy, such that pentobarbital inhibition must overcome a greater nerve output with normal sensitivity. To examine these possibilities, we first compared the slope of the amplitude dose/response curve of each condition as an indication of the effectiveness of pentobarbital. Condition had no significant effect on the slope of the amplitude dose/response curve. Our results are explained by a trend for burst amplitude to be increased during pregnancy (350 µV) compared to virgin (221 µV) and post-partum females (247 µV; p = 0.1).

The IC_50_ of pentobarbital on phrenic burst amplitude was 20.8 mg/kg in virgin females (95% CI, 18.7 to 23.3 mg/kg), 32.8 mg/kg in pregnant females (95% CI, 29.7 to 36.2 mg/kg), and 9.3 mg/kg in post-partum females (95% CI, 8.1 to 10.7 mg/kg)(Fig. 1*D*). The hill slope of the pentobarbital dose/response curve during pregnancy was −2.1±0.2 for amplitude, whereas the frequency hill slope was −9.4±1.8 (p<0.01). Both curves reach zero at the same dose, indicating that the inhibition of frequency is late-onset and rapid, whereas amplitude inhibition is early onset and gradual. In summary, burst amplitude was decreased similarly by sublethal doses of pentobarbital in all conditions, yet phrenic burst frequency was preserved at high doses of pentobarbital only during pregnancy.

### Hypercapnic-hypoxia challenge

Once phrenic motor output arrested due to barbiturate accumulation, we investigated the potential to restore respiratory activity with robust chemoreceptor stimulation via hypercapnic-hypoxia. 100 mg/kg of pentobarbital was sufficient to depress phrenic nerve activity in all groups (male, virgin female, pregnant, post-partum). Statistically, there was no difference in the incidence with which pregnant females and other groups responded to the hypercapnic/hypoxic stimulus. There was a non-significant trend for pregnant females to respond more frequently than other groups: 4 of 5 of pregnant rats responded *vs.* 2 of 6 virgin and only 1 of 6 post-partum rats (Fig. 1*E*). In contrast, 0 of 6 males responded to hypercapnic-hypoxia (data not shown) (Fisher's exact test; p = 0.1). In rats that responded, burst amplitude was 12.5% of baseline (pregnant), 1.7% (post-partum) and 6.4% (virgin female). In rats that did respond, burst frequency was 90% of baseline (pregnant), 199% of baseline (post-partum), and 98% of baseline (virgin).

### In vitro recordings

We hypothesized that the insensitivity of phrenic burst frequency to pentobarbital in pregnant rats resulted from altered GABA_A_R subtype expression in respiratory rhythm generating networks of the VRC. Thus, we examined cortical neurons as a control (Fig. 2*A*) and the medullary regions that contain neurons responsible for respiratory rhythm generation in the three slices through the VRC (Fig. 2*B,C*) *in vitro*. Neuronal activity in the VRC of male, virgin female, pregnant, and post-partum rats was initially categorized as: rhythmically bursting (rapid trains of ≥5 actions potentials separated by silent periods of equal duration; <10%, Fig. 2*D* top and bottom), fast tonic (>1 hz; ∼60%, Fig. 2*E* top), and slow tonic (<1 hz; ∼40%, Fig. 2*E* bottom). During the stabilization period, all rhythmically bursting cells converted into tonically active cells. No statistically significant differences were found in neuronal activity in subregions of the VRC (rVRG, preBötC, BötC) in any of the groups examined during baseline or after pentobarbital application (p>0.05). Neuronal activity was normalized to the mean of the 30 min baseline preceding drug application.

**Figure 3:**
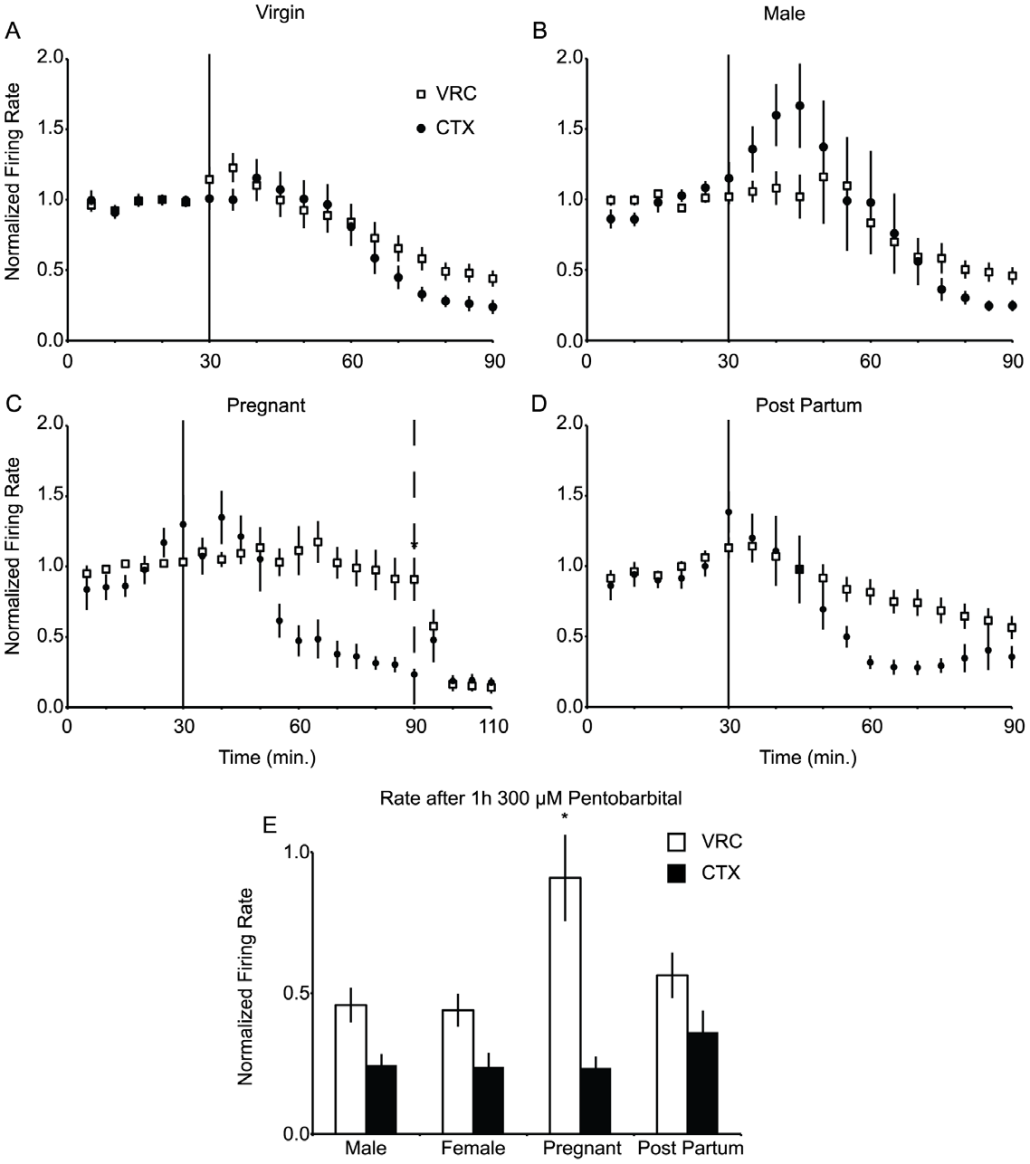
Neuronal responses to pentobarbital are altered in the medulla but not cortex of pregnant animals. Spontaneous activity of neurons in the VRC including rVRG, PreBötC, and BötC are insensitive to pentobarbital (300 µM) during pregnancy. ***A,*** Mean normalized firing rate of VRC (n = 37 closed circles) and CTX (n = 47, open squares) neurons from virgin female rats (n = 5 animals) in response to 300 µM pentobarbital (applied at vertical bar, t = 30 min). ***B,*** Pentobarbital had equivalent effects on VRC (n = 62) and CTX (n = 37) neurons from male rats (n = 6 animals). ***C,*** Pentobarbital (solid vertical line) failed to depress spontaneous activity of VRC neurons (n = 31) from pregnant animals (n = 4), yet inhibited CTX neurons (n = 26) in the same animals. Neuronal activity was modulated by barbiturate insensitive GABA_A_Rs, as 20 µM muscimol (dashed vertical line) rapidly silenced VRC neurons. ***D,*** This effect is reversible as both VRC (n = 51) post partum animals (n = 4) were inhibited by pentobarbital. ***E,*** Quantification of the normalized spontaneous activity of CTX (filled bars) and VRC (open bars) after 60 min of pentobarbital (300 µM) application. Single unit activity was normalized to mean baseline activity for that neuron (time 0–30 min). * denotes significantly different from virgin female. p<0.05.

In slices from virgin females (n = 5 rats), 1 h exposure to 300 µM pentobarbital inhibited spontaneous activity in both CTX (n = 47 neurons, 23.8±5% of baseline; p<0.05) and VRC (n = 37 neurons, 43.9±6% of baseline; p<0.05) (Fig. 3*A,E*) *vs.* time controls (n = 5 rats, 56 VRC neurons and 48 CTX neurons, 136±19% and 123±19% of baseline, respectively). Responses to pentobarbital of VRC (n = 62) and CTX (n = 37) neurons in slices from male rats (n = 6 rats) were similar to those of virgin females (Fig. 3*B,E*)

In slices from pregnant rats (n = 4 rats) 1 h exposure to 300 µM pentobarbital failed to alter the rate of spontaneous activity of VRC neurons (n = 31 neurons, 91±15% of baseline; p<0.05), yet the response of CTX neurons (n = 26 neurons, 23±4% of baseline) was equivalent to that observed in virgin females (p>0.05).

The persistence of spontaneous neuronal activity in the VRC of pregnant rats could reflect a lack of GABA_A_Rs on VRC neurons, or the presence of pentobarbital insensitive GABA_A_Rs in the same network. To distinguish these possibilities, we added 20 µM muscimol, a selective GABA_A_R agonist, to the aCSF bathing slices from pregnant rats after 1 h of 300 µM pentobarbital. After 1 h of exposure to 300 µM pentobarbital, VRC neurons from pregnant rats were rapidly (<5 min) inhibited by muscimol (14±4% of baseline; Fig. 3*C*). The response to muscimol of VRC neurons from pregnant rats did not differ from the response to muscimol of VRC neurons from time control rats (p>0.05).

The induction of pentobarbital insensitivity in VRC neurons during pregnancy was not sustained after pregnancy, as slices from 30 d post-partum rats (n = 4 rats) exposed to 1 h of 300 µM pentobarbital demonstrated significant inhibition of spontaneous activity in VRC neurons (n = 51, 56±8% of baseline; p<0.05 compared to pregnant VRC;) (Fig. 3*D,E*).

**Figure 4:**
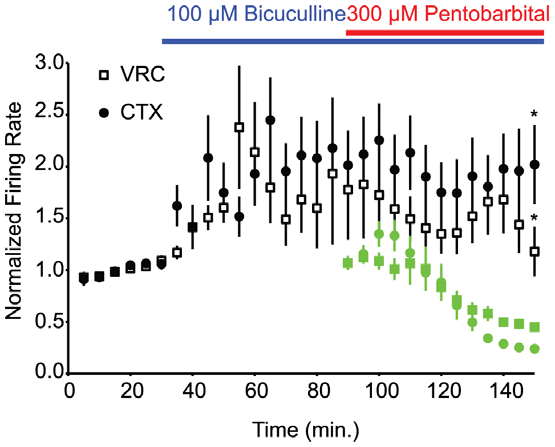
The GABA_A_R antagonist bicuculline prevents pentobarbital mediated inhibition of cortical and medullary neuronal activity. Spontaneous activity of VRC (n = 45 neurons from 4 rats, filled circles) and cortical (n = 59 neurons from 4 rats, open squares) before bicuculline, and during bicuculline (blue line) and pentobarbital coapplication (blue and red lines). Individual units are normalized to the 30 min baseline preceding bicuculline treatment. The corresponding VRC neuronal activity during 300 µM pentobarbital application (Fig. 3) is overlaid for comparison (light green trace). * denotes different than pentobarbital treatment, p<0.05.

To examine whether GABA_A_Rs mediated the primary inhibitory actions of 300 µM pentobarbital, we applied 100 µM bicuculline, a GABA_A_R antagonist, to slices (n = 4 rats, 2 males, 2 females, 45 [VRC] and 59 [CTX] neurons) prior to coapplication of 100 µM bicuculline and 300 µM pentobarbital. Bicuculline prevented the inhibition of spontaneous activity of both CTX and VRC neurons observed in virgin female and male slices (Fig. 4).

### GABA_A_R ε subunit mRNA

**Figure 5:**
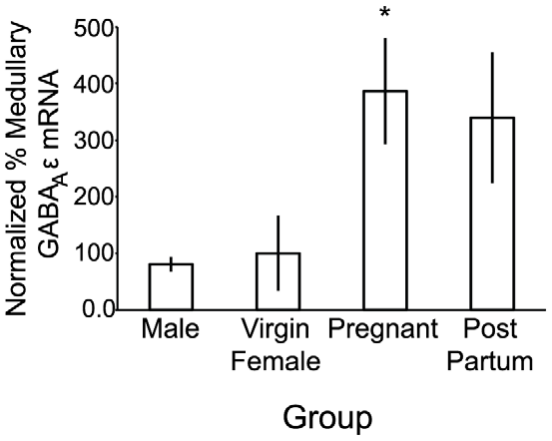
mRNA for the GABA_A_R ε subunit is increased in the medulla during pregnancy. Quantitative analysis of mRNA for GABA_A_R ε subunit in the medulla of virgin female, male, G17 pregnant, and 20–30 d post-partum rats. Medullary GABA_A_ ε mRNA was nearly 4-fold greater in pregnant than virgin female or male rats (p<0.05). Medullary GABA_A_R ε mRNA appeared elevated in post-partum rats, but the difference was not statistically significant compared to virgin females. For graphical representation, delta delta CT values were normalized to virgin females. Statistics were conducted on delta CT values. * denotes different than virgin female, p<0.05. Error bars indicate S.E.M.

We examined whole-medulla lysates for GABA_A_R ε subunit mRNA in male, virgin female, G17 pregnant, and 15–20 d post-partum rats (Fig. 5). Pregnant rats exhibited a nearly 4-fold increase in GABA_A_R ε subunit mRNA compared to virgin females (p<0.05). GABA_A_R ε subunit mRNA in post-partum rats exhibited a nonsignificant trend to increase (3.4-fold) *vs.* virgin females (p = 0.087).

### Immunohistochemistry

**Figure 6:**
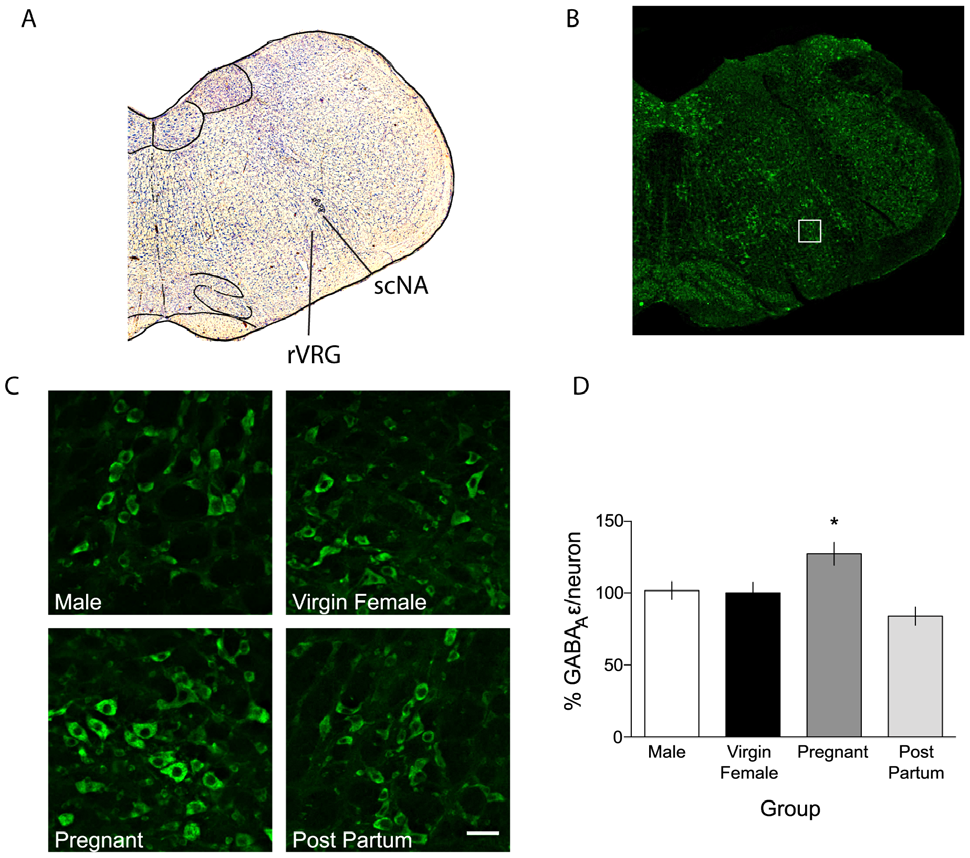
GABA_A_R ε subunit expression is increased during pregnancy in a region consistent with the rVRG and preBötC. ***A,*** Nissl stained coronal section demonstrates anatomical location of images previously stained for semi-quantitative immunofluorescence in relation to the NA. Nuclei in the Nissl stained section were identified with reference to the rat brain atlas (Paxinos and Watson, 2004). ***B,*** Fluorescence image of the section shown in ***A*** **,** reacted for GABA_A_R ε subunit (green). The white box is placed over the region of interest imaged at 40× for quantification. ***C,*** Sample images from male, virgin female, G17 pregnant, and 30 d post-partum rats showing GABA_A_R ε subunit immunoreactivity. ***D,*** Quantification of integrated density of cellular label in sections from male, virgin female, G17 pregnant, and post-partum rats. * denotes significantly different than virgin female, p<0.05. Scale bar is 50 µm. NA, nucleus ambiguus; scNA, subcompact Nucleus Ambiguus.

The GABA_A_R ε subunit was expressed abundantly throughout the medulla in all conditions, including robust label in the location of electrode placement during *in vitro* recordings, a region consistent with the rVRG and preBötC (Fig. 6*A,B*). The predominant pattern of staining in the VRC was in the cytoplasm of neurons and in primary neuronal processes (Fig. 6*C*). GABA_A_R ε immunofluorescent signal intensity in the VRC of pregnant rats was 127±8% of virgin females (baseline = 100±7.5%; p<0.05)(Fig. 6*D*). GABA_A_R ε immunofluorescence was equivalent between virgin females, and males (101.6±6% of virgin female). Post-partum females (83.9±6% of virgin female) were not statistically different than either males or virgin females. The GABA_A_R ε subunit was undetectable above background label in CTX slices.

The overall increase in GABA_A_R ε immunoreactivity in medullary sections containing the rVRG and preBötC during pregnancy could be explained by (1) an increase in the number of positive cells, (2) an increase in the intensity of positive cells, or (3) a combination of number and intensity increases. Our data are largely explained by an increase in the mean signal intensity of individual neurons (116±4% of virgin female; p<0.05). There was no significant increase in either the number of GABA_A_R ε positive cells in slices through the VRG (mean number of positive neurons, unihemispherically; 137.75±14.37 male, 107.25±33.42 virgin female, 114.75±27.05 post partum, 127.±32 pregnant) or the size of GABA_A_R ε positive cells during pregnancy.

### NK1-receptor and GABA_A_R ε immunoreactivity are co-expressed in the VRC

**Figure 7:**
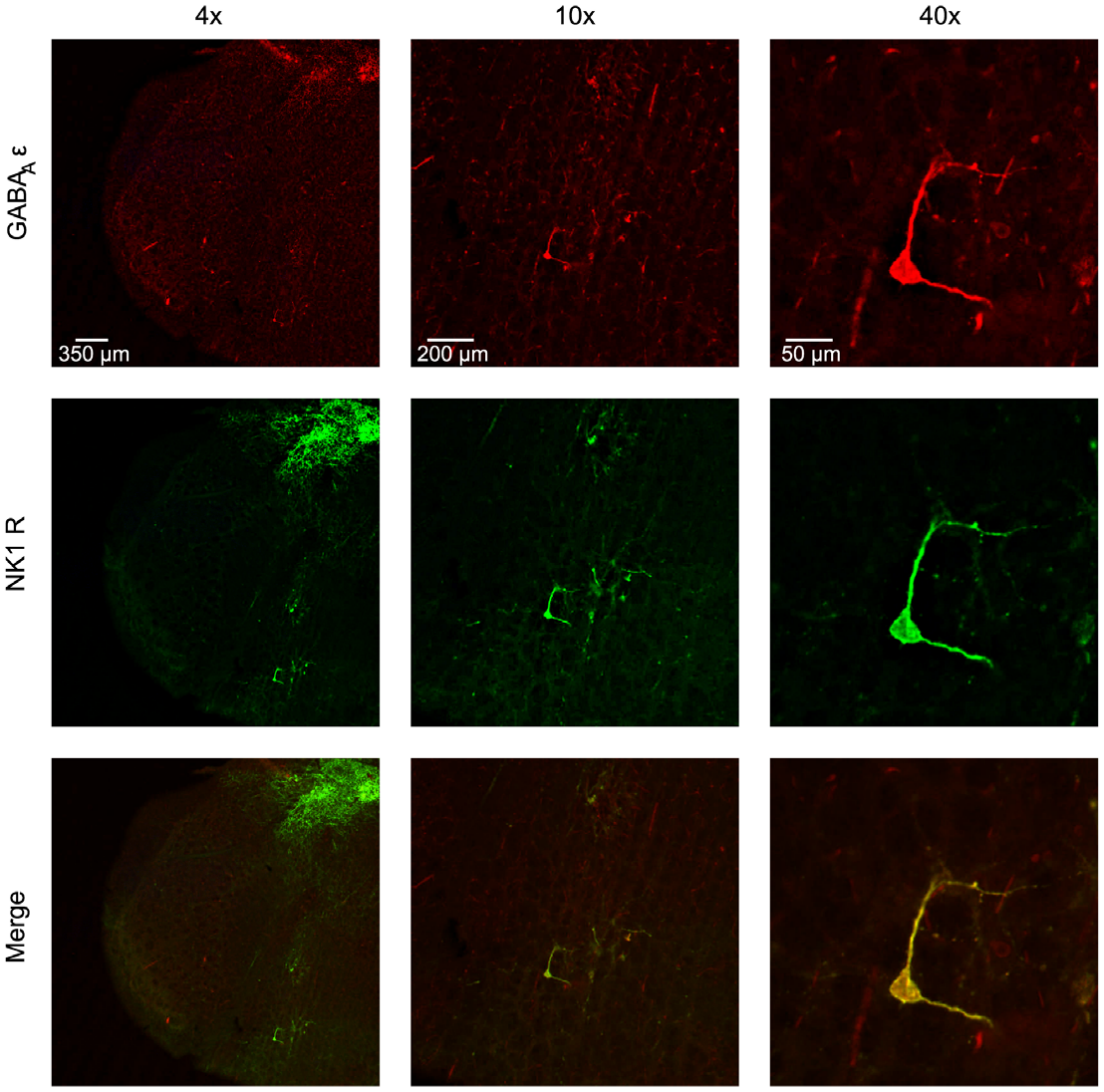
GABA_A_ ε is expressed on putative respiratory neurons that are pentobarbital insensitive during pregnancy. Slices previously used for *in vitro* electrophysiology were sectioned and reacted against the GABA_A_R ε subunit (red, top) and NK1-R (green, middle). NK1-R is a marker of respiratory neurons in the preBötC and ventral respiratory column (VRC). Images were acquired in the location of electrode placement. The GABA_A_R ε subunit is expressed on NK1-R positive neurons in the preBötzinger Complex of the medulla (merge, bottom). Example image was acquired in the preBötzinger Complex of a virgin female rat.

We examined slices used in *in vitro* experiments immunohistochemically to confirm that: 1) *in vitro* recordings were conducted on a population of neurons in three respiratory regions of the VRC (rVRG, preBötC, BötC) including those believed to be critical for rhythm generation (preBötC), and 2) the GABA_A_R ε subunit is expressed on putative respiratory neurons in these regions. Sections were co-labeled for the GABA_A_R ε subunit and for the Substance P receptor, NK1-R, which primarily labels respiratory neurons in the rVRG and preBötC [24], [25]. NK1-R and the GABA_A_R ε subunit exhibited extensive colocalization, both in the NA (subcompact and compact), which was used as a landmark for electrode placement, and ventral to the NA, consistent with VRC neurons (Fig. 7). All NK1-R positive neurons in the VRC demonstrated GABA_A_R ε subunit immunoreactivity, yet not all GABA_A_R ε subunit positive neurons showed NK1-R immunoreactivity. While these ε subunit positive neurons surrounded the electrode placements, it is impossible to confirm the specific identity of cells recorded in electrophysiological experiments. Further, due to the nature of *in vitro* electrophysiological data collection, the slices used to collect these data were harvested individually and at different points in time, making attempts at quantitative or qualitative comparisons inappropriate.

## Discussion

Here we describe a novel form of natural, reversible plasticity in the respiratory system during pregnancy. In pregnant rats, phrenic motor output is highly resistant to depression by pentobarbital, a positive allosteric modulator of GABA_A_Rs. We suggest that the mechanism underlying respiratory insensitivity to pentobarbital during pregnancy is an upregulation of the GABA_A_R ε subunit, which renders GABA_A_Rs insensitive to pentobarbital, and other positive allosteric modulators. At least one locus of increased ε subunit expression relevant to the preservation of respiratory function is neurons in the medullary ventral respiratory column (VRC). Both phrenic motor output in anesthetized rats (*in vivo*) and VRC neuronal activity (*in vitro*) are insensitive to pentobarbital during pregnancy and display increased GABA_A_R ε subunit mRNA and protein levels. The GABA_A_R plasticity described here is regionally specific since cortical neurons remain highly sensitive to pentobarbital during pregnancy. Although GABA_A_R plasticity is centered in the VRC, we cannot exclude additional changes in other medullary or spinal neurons that control breathing. We hypothesize that these findings will extend to other allosteric modulators, including endogenous neurosteroids, whose levels increase dramatically during pregnancy. Further work is required to establish the full pharmacological range and implications of this plasticity.

Respiratory rhythm generation arises from the emergent network activity of a small population of neurons in the rostral ventrolateral medulla [26]–[28]. While we aimed to record from this population of neurons, it is important to recognize that the *in vitro* data presented here were collected from putative respiratory neurons lacking functional identification. Some of the individual neurons recorded were almost certainly not respiratory-related, yet we are confident that the mean effects that emerged from our data are related to respiratory function due to three facts; first, our electrodes sampled a large number of isolatable units in a region anatomically consistent with the ventral respiratory column. While not all of the units recorded were respiratory-related, it is unlikely that we failed to collect data from a large number of respiratory neurons. Second, our *in vitro* data reflect our *in vivo* measurement of phrenic nerve activity. Third, a subpopulation of the neurons recorded *in vitro* demonstrated transient patterns of spontaneous activity consistent with previous reports of respiratory neurons *in vitro* [29]–[31], [26]. With this in mind, interpretation of the *in vitro* data presented here must be careful. Conservatively, we demonstrated that during pregnancy, a large population of neurons in the ventrolateral medulla is insensitive to doses of pentobarbital that typically inhibit neuronal activity in all brain regions investigated. It is tempting to assume that these neurons are directly responsible for the parallel finding in our *in vivo* phrenic nerve recordings, but a direct, causal link must first be established. Two additional findings further suggest that the *in vitro* physiology data were recorded in populations of respiratory neurons. First, immunohistochemical analyses of slices used for recordings demonstrate that electrodes were centered in a region containing NK-1 receptor expressing neurons (the NK-1 receptor is a marker of respiratory neurons in the preBötC) [24], [25]. Second, NK-1 receptor immunopositive neurons were colocalized with the ε-subunit in pregnant rats. Taken together, these data suggest but do not confirm a direct connection between the pentobarbital-insensitivity of putative respiratory neurons in the VRC and the insensitivity of phrenic nerve burst frequency to high doses of pentobarbital. Further work will be needed to unequivocally demonstrate the mechanism underlying these data.

Two alternative explanations for respiratory insensitivity to pentobarbital during pregnancy merit discussion. The first alternate explanation is that pentobarbital may cross the hemato-placental barrier, thus altering levels of pentobarbital available to the central nervous system of the mother compared to non-pregnant animals. Two components of our data suggest that this is not the case. First, our slice data indicate that this plasticity is apparent at the level of medullary respiratory control. This *in vitro* approach eliminates the potential for the placenta to absorb pentobarbital that would otherwise enter the CNS. Second, in the *in vivo* recordings, pentobarbital had a robust effect on other CNS functions, such as blood pressure, regardless of pregnancy-state (the best-fit 2^nd^ order polynomial of normalized virgin MAP across doses of pentobarbital was y = −0.0081x^2^+0.061x+0.9094, while pregnant was y = −0.0043x^2^+0.0235x+0.8825). Together, these data reveal that pentobarbital has equivalent access to the CNS in both pregnant and non-pregnant animals, and that the resistance to depression observed in the respiratory system is somewhat specific to that modality. The second alternate explanation of our data is that respiratory resistance to pentobarbital could simply reflect the deletion of GABA_A_Rs on respiratory neurons during pregnancy. This explanation would be consistent with baseline hyperventilation during pregnancy [32]. However, *in vitro* data strongly suggest that the respiratory system does not delete GABA_A_Rs, since all recorded neurons remained highly sensitive to muscimol, a GABA_A_R agonist. Taken together, our electrophysiological (*in vivo* and *in vitro*), protein, and mRNA data strongly support increased native expression of the GABA_A_R ε subunits in the medulla in pregnant rats. Our finding of neurons with fully functional GABA_A_Rs that are insensitive to pentobarbital is consistent with reported properties of transfected GABA_A_Rs containing ε subunits in model systems [12], [33].

### Phrenic nerve burst frequency and amplitude

*In vivo* data suggest that neurons responsible for phrenic burst frequency and amplitude are dissociable with respect to their pentobarbital sensitivity. Frequency became insensitive to pentobarbital during pregnancy whereas amplitude responses to progressively increasing pentobarbital doses were unaffected. This differential response is consistent with observed GABA_A_R ε subunit plasticity in the pre-Bötzinger Complex, since this anatomical region plays a major role in respiratory rhythm generation (*versus* pattern formation). Further, ε subunit plasticity in these neurons must be reversible since similar pentobarbital insensitivity was no longer observed in post-partum rats. On the other hand, GABA_A_R ε subunit plasticity in other VRC neurons, presumably involved in burst pattern formation, was expected to alter amplitude (tidal volume) responses to increasing doses of pentobarbital.

**Figure 8:**
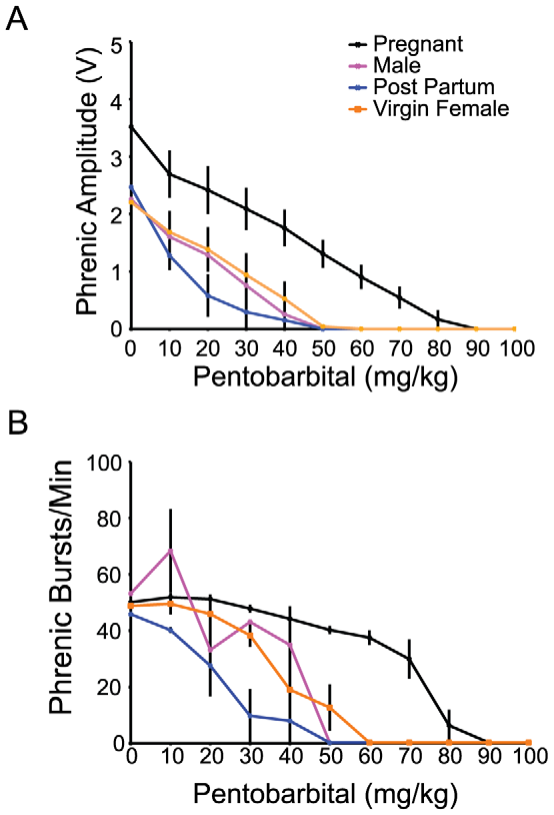
Phrenic nerve amplitude but not phrenic nerve burst frequency is sensitive to pentobarbital application during pregnancy. ***A,*** Phrenic nerve amplitude in response to pentobarbital *in vivo* is marginally increased at baseline during pregnancy compared to all other groups, but shows an equivalent rate of decline with increasing doses of pentobarbital. ***B,*** Phrenic nerve burst frequency in response to pentobarbital *in vivo* shows pregnancy-specific insensitivity to sub-lethal doses of pentobarbital compared to the incremental effects of dose on phrenic bust frequency in male, virgin female, and post-partum rats.

The differing effects of pentobarbital on burst amplitude versus frequency during pregnancy are complicated. First, our data indicate that the amplitude-generating network was equally sensitive to pentobarbital in all conditions since the slope of the phrenic amplitude *vs.* pentobarbital dose was not statistically different in any condition (Fig. 8*A*). Second, in any condition, the loss of phrenic bursts at high pentobarbital doses may simply represent our inability to detect very small bursts (as opposed to a slowing burst frequency). If the second point were correct, the slope of phrenic burst frequency vs. dose plot should remain constant across increasing pentobarbital doses (due to the lack of effect) when the burst amplitude is reliably measurable. At greater doses (when burst amplitude is at the threshold for detection) burst frequency will rapidly approach zero. Such a trend was observed only in pregnant rats (Fig. 8*B*) where phrenic burst frequency decreased minimally with increasing pentobarbital dose, and then dropped dramatically as phrenic amplitude reached zero. Thus, it is possible that during pregnancy, neurons responsible for rhythm generation are unaffected by the doses of pentobarbital used here; the lethal dose for burst frequency may actually reflect the effects of pentobarbital on burst amplitude.

A limitation of *in vivo* studies is that it is difficult to identify precisely the regional or molecular targets of pentobarbital. We predicted that phrenic nerve burst frequency plasticity during pregnancy arose from a plasticity in medullary GABA_A_Rs on neurons involved in respiratory rhythm generation. Thus, we isolated the region most often associated with respiratory rhythm generation (ie. the preBötC) to investigate network-specific plasticity that may contribute to the *in vivo* phenotype. Slices for the *in vitro* experiments also included the BötC, which mediates powerful synaptic inhibition during expiration [34]. Our data indicated no detectable rostro-caudal differences in the pentobarbital sensitivity of VRC neurons, suggesting that GABA_A_R plasticity may be characteristic of all brainstem respiratory neurons. Indeed, GABA_A_R ε subunit expression did not differ between the rVRG and the region of the preBötC.. Although all neurons recorded were not rhythm-generating neurons of the PreBötzinger Complex, the electrode array was sufficiently large that it contained at least some of this neuronal population. The observation that nearly all medullary neurons recorded had similar properties and responses to pentobarbital provides strong support that the concepts described here pertain to respiratory rhythm generating neurons in pregnant rats.

While we have demonstrated a significant role for compensatory regulation of ε subunit in medullary respiratory networks in pregnant rats, other GABA_A_R subtypes may contribute to this plasticity. For example, a decrease in δ subunit-containing GABA_A_Rs, which heighten sensitivity to neurosteroids and pentobarbital [35], [36], might amplify the role of the ε subunit during pregnancy. To prevent excessive inhibition, the upregulation of a tonic current-conducting GABA_A_R population (ε subunit containing GABA_A_Rs) mandates the simultaneous down-regulation of other GABA_A_Rs. While we did not investigate downregulation of constitutively expressed GABA_A_Rs, previous reports suggest decreased hippocampal γ_2_ subunit-containing GABA_A_Rs coincident with changes of δ subunit-containing GABA_A_Rs during pregnancy [16]. Compensatory changes in other subunits are implied by the observation of hyperventilation during pregnancy [9], [32], further indicating that the respiratory system is not under excessive inhibition during gestation.

It is worth noting that this compensatory plasticity is probably more complex than our data is able to reveal. Specifically, post-partum females often failed to demonstrate statistically significant differences from virgin animals, but the data reveal biologically interesting trends that suggest a persistent change in respiratory control after parturition.

For instance, in Fig. 1, post-partum females demonstrate a visible trend of heightened pentobarbital sensitivity compared to virgin females. In Fig. 6*D*, the % GABA_A_ ε/neuron for post-partum females (83.9 ą 6% of virgin female) falls below the value of virgin female and male (101.6 ą 6% of virgin female) rats. In Fig. 8, the slopes for post-partum females are nominally below the values of the virgin female and male rats. Our results support the hypothesis of a reversible mechanism, but also indicate the possibility of a rebound effect after pregnancy. This matter is not clear-cut, however, as in Fig. 5 the normalized medullary GABA_A_ ε mRNA for post-partum females is closer to the values of pregnant females than other groups. We speculate that posttranscriptional control of the GABA_A_ ε subunit significantly affects expression patterns.

### GABA in respiratory control

Bicuculline application to VRC neurons reveals a tonic GABA_A_R current that imparts a degree of GABA-mediated gain modulation to the respiratory system [3]. However, the molecular components of this GABA-mediated gain modulation have yet to be identified. Tonic GABA_A_R currents are frequently associated with extrasynaptic, δ subunit-containing GABA_A_Rs [37], [38], [39]. These GABA_A_Rs are highly sensitive to modulation by neurosteroids [36], including neurosteroids that increase during pregnancy. In the hippocampus, the pregnancy-related augmentation of this tonic current caused a homeostatic down-regulation of δ subunit-containing GABA_A_Rs in the hippocampus, thereby maintaining neuronal excitability [15]. The data reported here suggest that ε subunit-containing GABA_A_Rs may represent a form of compensatory plasticity in the gain control system of the respiratory rhythm generating neurons in the VRC, similar in some respects to GABA_A_R plasticity in the hippocampus [15]. The rare ε subunit-containing GABA_A_Rs is also upregulated during hibernation in the rostral ventrolateral medulla of 13-lined ground squirrels [11]. ε subunit-containing GABA_A_Rs are insensitive to allosteric modulation by neurosteroids, and may mediate an extra-synaptic tonic current [13].

Molecular changes in GABA_A_R subunits in the respiratory rhythm-generating network are difficult to quantify precisely. However, the nearly 4-fold increase in ε subunit mRNA in the medulla in pregnant rats indicates that there is significant transcriptional regulation of this subunit during pregnancy. Our immunohistochemical data showed that one site of increased protein expression in the medulla is in the VRC. Since all identified NK1-R positive neurons expressed ε subunit, but not all the ε subunit positive cells expressed the NK1-R, GABA_A_Rs with ε subunits may be regulated differently in different populations of medullary neurons. Alternately, if the increase in ε subunit expression were restricted to respiratory neurons, unchanging NK1-R negative/ε subunit positive neurons would have diluted our ability to detect increased subunit protein levels. However, since mRNA increased in whole-medulla lysates, multiple medullary networks may regulate ε subunit-containing GABA_A_Rs during pregnancy. Since many critical homeostatic neural networks reside in the medulla, ε subunit-containing GABA_A_Rs upregulation may also protect these vital physiological functions from neurosteroid inhibition during pregnancy.

### Regulation of GABA_A_R subtype expression

**Figure 9:**
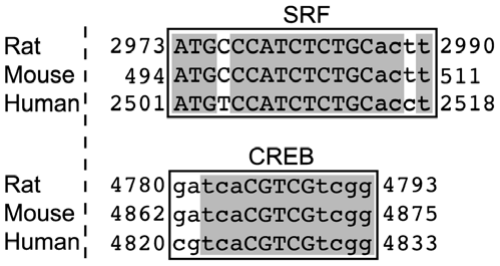
The 5′ flanking region of the GABA_A_R ε gene exhibits highly conserved transcription factor binding sites in rat, mouse, and human that are consistent with activity-dependent gene expression. Rat, mouse and human GABA_A_R ε genes were analyzed for putative transcription factor binding sites using the NCBI program MULAN. Within the first 5 kb of the 5′ flanking region of this gene, we identified a putative binding site for the Serum Response Factor (SRF) and the Cyclic AMP Response Element Binding protein (CREB) which are 96 and 95% identical, respectively, among the rat, mouse and human genes. Starting and ending basepair location relative to the 5 kb 5′ flanking region are listed to the left and right of each sequence.

During pregnancy, we speculate that increased progesterone levels regulate GABA_A_R subtype expression patterns. For example, circulating neurosteroids could decrease the I/O ratio of respiratory rhythm-generating neurons early in gestation, thereby inducing gene expression due to decreased neuronal activity. Using the NCBI program MULAN (http://mulan.dcode.org), we identified at least two transcription factors with consensus binding sites in the 5′ flanking region of the gene encoding the GABA_A_R ε subunit in rats, mice, and humans (Fig. 9). The first transcription factor, cyclic AMP response element binding protein (CREB), is critical for multiple forms of activity dependent neuronal gene expression and neuronal plasticity [40]. The second, serum response factor (SRF), mediates expression of many neuronal genes [41], including transcription of Arc/Arg3.1 [42], a key regulators of homeostatic synaptic plasticity [43]. Alternatively, sex steroid hormones and their metabolites may exert direct genomic effects on neurons that do not depend on activity *per se* [44]. At this time, specific mechanisms linking pregnancy with increased ε subunit-containing GABA_A_Rs remain unknown.

### Significance

Here we provide the first description of a novel, physiologically relevant form of plasticity in the respiratory control system. This plasticity involves the expression of a relatively rare yet conserved GABA_A_R subunit that confers neurosteroid, anesthetic, ethanol, barbiturate, and benzodiazepine insensitivity [11]–[13]. These data are consistent with a compensatory plasticity that is reversible since barbiturate responses and ε subunit protein levels return to normal once the pregnancy has ended and neurosteroid levels return to normal. Our results suggest that pregnant female rats reorganize neural functions associated with the control of breathing by utilizing a mechanism similar to that observed in hibernating ground squirrels [10], [11], achieving a more resilient respiratory motor output during a critical period of life, reproduction. Taken together, these findings suggest an evolutionarily conserved mechanism for controlling the interaction of endogenous allosteric modulators of GABA_A_Rs (e.g., hormone metabolites) and breathing.

Our findings also have potential clinical relevance in to the response of pregnant women to general anesthetics or anxiolytics used during surgery or post-operative care. Pregnant women may be somewhat protected against cardiorespiratory depression if these agents are used. On the other hand, these (presumably) adaptive responses may also provide inadvertent cardio-respiratory protection against some drugs of abuse, including ethanol [11].
